# Measuring COVID-19 anxiety among russians: Examining the psychometric properties of russian translations of the covid-anxiety scale and the fear of coronavirus-19 scale

**DOI:** 10.1192/j.eurpsy.2021.1366

**Published:** 2021-08-13

**Authors:** C.A. Lewis, E. Sinelnikova, J. Malik

**Affiliations:** 1 Department Of Social And Behavioural Sciences, School Of Social And Health Sciences, Leeds Trinity University, Leeds, United Kingdom; 2 Psychology, St. Petersburg State Transport University, St. Petersburg, Russian Federation; 3 Psychology, Quaid-i-Azam University, Islamabad, Pakistan

**Keywords:** COVID-19, Anxiety, Russian, translation

## Abstract

**Introduction:**

Both the COVID-Anxiety Scale and the Fear of Coronavirus-19 Scale have been recently developed to facilitate research on COVID-19 anxiety.

**Objectives:**

To examine the psychometric properties of Russian translations of the COVID-Anxiety Scale and the Fear of Coronavirus-19 Scale.

**Methods:**

In order to examine the psychometric properties of Russian translations of the COVID-Anxiety Scale and the Fear of Coronavirus-19 Scale, a total of 341 Russian adults completed both measures.

**Results:**

First, a high level of COVID-19 anxiety was found in the sample. Second, confirmatory factor analysis demonstrated that the Russian translations of both the COVID-Anxiety Scale and the Fear of Coronavirus-19 Scale had satisfactory psychometric properties, with both scales having a hypothesised one-factor structure. Third, a significant positive association was found between both the COVID anxiety scales. Fourth, higher COVID anxiety scores were associated with being female, and being older.

**Conclusions:**

These findings provide initial evidence for the satisfactory properties of the Russian translations of the COVID-Anxiety Scale and the Fear of Coronavirus-19 Scale. Further research is suggested that examines the prevalence and psychological correlates of COVID-19 anxiety.

